# A study on the belt and road initiative’s trade and its influencing factors: Evidence of China-South Asia’s panel data

**DOI:** 10.1371/journal.pone.0282167

**Published:** 2023-04-14

**Authors:** Ling Zhou, Yanghai Mao, Qinyi Fu, Danlu Xu, Jiaqi Zhou, Shaolong Zeng

**Affiliations:** 1 School of Marxism, Hangzhou Normal University, Hangzhou, China; 2 School of Finance and Economics, Xizang Minzu University, Xianyang, China; 3 School of Economics, Hangzhou Normal University, Hangzhou, China; 4 Institute of Scientific and Technical Information, Chinese Academy of Tropical Agricultural Science, Haikou, China; 5 Hangzhou Economist Association, Hangzhou, China; University of Pecs Faculty of Humanities: Pecsi Tudomanyegyetem Bolcseszettudomanyi Kar, HUNGARY

## Abstract

The Belt and Road Initiative (BRI) is a development plan proposed by China that aims to build a new platform for international cooperation and create new drivers of shared development. South Asia is a key area in the Belt and Road Initiative, including eight countries. As the BRI implemented, China’s trade with South Asia has been gradually strengthened. This paper explores the influencing factors of China-South Asia trade under the background of the BRI by using Gravity Model of Trade. The results show that economic growth in China and South Asia, increase of savings rate and improvement of industrialization in South Asia has a significant positive effect on China-South Asia trade. While the development gap between China and South Asia has negative effect on China-South Asia trade.

## 1. Introduction

The Belt and Road Initiative (BRI) was first proposed by Chinese President Xi Jinping in 2013 [1]. The Belt is a land-based route leading from China to Europe via Central Asia. And the Road is its sea-based equivalent, passing through Southeast Asia, Africa and the Middle East on its way to Italy. BRI has received support and response from many countries. By October 2019, 137 countries and 30 international organizations signed 197 cooperation documents with China [2]. Connectivity through the BRI should be created by combining the building of infrastructure, the development of stronger regulations and the promotion of more personnel exchanges. BRI not only promotes economic cooperation between China and the world, but also promotes the transformation of the global governance system and speeds up China’s opening-up.

South Asia is a key area in the BRI, including Afghanistan, Bangladesh, Bhutan, India, Maldives, Nepal, Pakistan, Sri Lanka. With the construction of Economic Corridor in South Asia, including China-Pakistan Economic Corridor (CPEC), Bangladesh-China-India-Myanmar Economic Corridor (BCIM) and China-Nepal-India Economic Corridor, the trade cooperation between China and South Asia has been gradually strengthened. South Asia has unique geographical advantages in linking the east and the west and connecting the land and sea. Although the contribution of South Asia in the global economy is relatively low, it is the fastest-growing region in Asia-Pacific Region, with an economic growth rate of 7.6% from 2014 to 2018. The study on China-South Asia trade and the factors affecting it under BRI will help improving trade cooperation between China and South Asia and promoting the healthy development of BRI in South Asia.

The rest of paper is organized as follows. Section 2 provides the review of the literature. The trend and structure of China-South Asia trade are discussed in Section 3. Section 4 contains the theoretical framework and methodology. The empirical results are presented in Section 5. Section 6 contains conclusion and policy recommendations.

## 2. Literature reviews

### 2.1. BRI and trade interdependence

There are many researches on the opportunities and challenges of trade between China and South Asia. Tong & Yi (2019) assess the BRI’s effect on China’s trade development and find that there are huge variations across regions within the Belt and Road countries in their trade relations with China [3]. Du (2017) sum up the progress of the BRI construction in South Asia and the characteristics of international cooperation under the background of BRI in South Asia [4]. Xu et al. (2018) analyze the development and dilemma of China-South Asia regional cooperation through the "Five Links" of BRI [5]. Ramasamy & Yeung (2019) compare the impact of the main initiatives of the One Belt One Road (OBOR), and find overwhelming evidence that shows improvements in border administration has the greatest impact on exports of corridor countries [6]. Hu et al. (2017) suggest the main factors that restrict the trade between China and South Asia including the unstable political situation of some countries in South Asia [7].

Trade interdependence between China and South Asia is also studied by many scholars. Zhao et al. (2019) use Hubness Measurement (HM) Index to measure the trade interdependence between China and South Asia. The results show that the dependence of China’s exports on South Asia countries is significantly lower than the dependence of South Asia countries’ exports on China. Since BRI was proposed, the overall dependence of China’s exports on South Asia countries has been increasing, while the dependence of South Asia countries’ exports on China is declining [8]. Chen & Xu (2018) analyze the trade complementarity and competitiveness between China and South Asia by measuring indicators such as revealed comparative advantage (RCA) index, export similarity index (ESI), trade integration index (TII) and complementarity index (CI). They find that both competitiveness and complementarity exist in the trade between China and South Asia countries. China mainly exports manufactured goods to South Asia countries, while South Asia countries mainly export primary and semi-finished goods to China [9]. Li & Lei (2019) calculate the trade competition index and trade complementarity index between China and four South Asia countries. The results show that China’s trade with Pakistan, Sri Lanka and Maldives are partial complementary, while China-India trade is both competitive and complementary [10]. Hu et al. (2017) study the trade competitiveness and complementarity of trade between China and South Asia countries on different products by calculating RCA and trade complementarity index (TCI). They suggest that China has an absolute advantage over South Asia countries in capital-intensive and technology-intensive machinery and transportation equipment products, while South Asia countries have advantages in food and live animals, manufactured products. China-South Asia trade is both competitive and complementary in labor-intensive industrial manufactured goods trade. China and South Asia are highly complementary in food, live animals and minerals [7]. Feng & Liu (2017) study the China-South Asia trade by analyzing comparative advantage and competitive advantage of China and South Asia. The industrial international competitiveness of 9 industries in China and South Asia countries is studied. The results are consistent with Hu et al. [11]. Foo et al. (2020) explore the potential effects of OBOR policy on trade flows in ASEAN countries and China. They use the augmented gravity model of international trade and data on ASEAN countries and China from 2000 to 2016. The empirical results show that the coefficient of the OBOR dummy is positive and statistically significant, which implies that this policy benefits both ASEAN countries and China in terms of increased trade flows among these countries [12].

### 2.2. Trade and economic growth

Khan (2020) examines whether trade openness and inward FDI may affect income distribution in an unbalanced panel of five South Asian countries over the period of 1990–2016. The results reveal that trade and FDI have significant effects on income inequality, however, inverted U-shaped curve holds for trade as purposed by the trade theory [13]. Basnet (2020) examines the long-run impact of terms of trade (TOT) on economic growth in the context of eight South and Southeast Asian emerging economies. Economic growth over the last three decades has been impressive in the region though TOT has shown an overall declining trend. The results provided adequate evidence of the long-run relationship of TOT with income and investment in South and Southeast Asia. Despite a positive relationship, TOT movements did not exert a significantly large impact on income in the short run [14]. Rahman et al. (2020) investigate the impact of CO2 emissions, population density, and trade openness on the economic growth of five South Asian countries by using data from 1990 to 2017. The panel co-integration approach of extended neoclassical growth model is used. And the results reveal that CO2 emissions and population density positively and trade openness negatively affect the economic growth in South Asia [15]. Kumar (2020) presents the facts on India’s role in the economic development of South Asia region while testing the potential spillovers of India’s trade and economic growth. The results highlight that the economic growth and regional trade of India are found significant short and long run spillovers on the economic growth of Bangladesh, Sri Lanka, Nepal and Bhutan [16]. Zakaria et al. (2016) empirically examine the effects of trade liberalization on undernourishment and income inequality in South Asian countries (SACS). The estimated results reveal that undernourishment has decreased while income inequality has increased in the region after liberalization [17]. Tristan (2019) considers the effect of BRI on supply-chain trade for 64 economies. He employs a structural gravity equation to estimate the impact of trade-cost reducing measures notably infrastructural improvements and the creation of free trade agreements on supply-chain trade and welfare in general equilibrium. The results show that infrastructural investments will yield asymmetric benefits to China, Russia and Southeast Asian countries stemming from greater European market access [18].

### 2.3. Trade influencing factors

Huang et al. (2020) construct a measurement equation for China’s exports to the five Central Asian countries based on the gravity model of international trade for the purpose of forecasting China’s future export growth potential under the background of the BRI. They use panel data from 2010 to 2017 to perform multiple regression analysis under the random effects model. They find that the model using China’s GDP, trading partners’ GDP, geographical distance, and borders as explanatory variables has a higher degree of fitness and each key explanatory variable is significant [19]. Hu (2014) explores the factors influencing China’s exports to South Asia countries using Gravity Model, which includes GDP of China and host countries, FDI inflows of South Asia, nominal exchange rate of RMB against the US dollar, geographical distance, the degree of openness of South Asia [20]. Xu & Liu (2019) also investigate the factors affecting the trade between China and BRI countries by using Gravity Model. The results show that economic development, market size, income, trade facilitation and FDI have effect on the trade [21]. Kong (2018) study the influencing factors of bilateral trade between China and 64 BRI countries. The results show that GDP growth of China and trade facilitation condition promote bilateral trade. While geographical distance, institutional distance and adjacency effect have negative effect on trade [22]. Han & Lv (2018) find out the improvement of trade facilitation can improve the terms of trade of China-South Asia trade [23].

There are other researches find out the trade can also be affected by intuitional and cultural factors. Ren & Liu (2017) study on the trade between China and Central Asia and find that the GDP of China and Central Asia countries, the population of host countries, China’s dependence on foreign trade and the accession to World Trade Organization (WTO) have positive effect on China-Central Asia trade [24]. Wan (2019) investigate the influencing factors of trade between China and 22 BRI countries. The results show that the accession to WTO, Asia-Pacific Economic Cooperation (APEC) and BRI have significant positive effect on the trade between China and BRI countries [25]. Zhang (2019) find that GDP, population, transportation distance, whether the two countries are bordered and the accession to the WTO, APEC, Shanghai Cooperation Organization, Asian Investment Bank have certain effect on the bilateral trade between China and BRI countries [26]. Li et al. (2019) explore the factors affecting the trade between China and 33 BRI countries based on Stochastic Frontier Gravity Model. They find that economic development, the population of importer, financial freedom, infrastructure, frequency of cultural exchanges can promote the bilateral trade, distance has a negative effect on it [27].

Akram (2020) analyzes the causes of low intra-regional trade connections within South Asia. By using World Bank data and other relevant sources for the period 1995–2018, he finds that the South Asian Free Trade Area (SAFTA) countries are not actually natural trading partners. Rather they are often competitors, seeking to export the same product groups [28]. Kumar (2020) applies trade intensity index of four largest South Asian countries (India, Bangladesh, Pakistan, and Sri Lanka) to estimate the short‐run and long‐run trade co‐integration in autoregressive multilateral framework. He finds that there are long‐run trade complementarities between the trade of Pakistan and Sri Lanka, while short‐run trade complementarities exist between India and Bangladesh, and between India and Sri Lanka. Pakistan and Bangladesh are found close trade competitors in South Asia in short run [29]. Mahmood & Jongwanich (2018) examine the effects of in-effect free trade agreements (FTA) on exports of Pakistan using the extended gravity model of bilateral trade flows. Their systematic comparison of both the measures of an FTA suggests that the estimation based on the tariff gap is consistent with the observed changes in the trade pattern of Pakistan. Pakistan-China FTA (PCFTA) has the largest stimulating effect for Pakistan’s exports, while the effects of other FTAs are much smaller and not much different from each other. The effects of FTAs on agricultural products tend to be higher than those of manufacturing ones, suggesting ability of firms in the former to better comply with imposed rules of origin (ROO) than the latter [30].

### 2.4. Effect on a certain type of goods

In addition to the research on the influencing factors of the overall bilateral trade flow, some scholars also study the influencing factors of the bilateral trade from perspective of one type of goods, such as agricultural products and digital trade.

Kumar (2021) use qualitative approach and quantitative analysis got the results establish the presence of agri-trade barriers from South Asian countries against India as well as India’s barriers against rest seven countries of South Asia [31]. Hatab et al. (2010) find that the fluctuations in economy, population, exchange rate have impact on agricultural exports by using Gravity Model [32]. Hong (2019) analyzes the situation and structure of agricultural trade between China and 5 Central Asia countries. The results show that GDP, population, difference of GDP per capita, distance, agricultural value added (% of GDP) and accession to the BRI could affect the agricultural trade between China and the five Central Asia countries [33]. Kaur et al. (2020) use revealed comparative advantage indices to assess the comparative advantage and the indicative trade potential of different South Asian countries in various services sub-sectors. The study reveals that there stand complementarities in the trade of services as Pakistan and Sri Lanka have a competitive advantage in Transport Services, while India has a competitive advantage in Computer and Information Services and Other Business Services. In travel services, Maldives and Nepal possess competitiveness, while Bangladesh in Government Services [34]. Tan et al. (2015) find that spatial distance, income gap, population, openness and are important factors affecting China’s agricultural exports to BRI countries [35]. Hu & Qi (2018) find that there is a large development potential and cooperation space for agricultural products trade between China and four South Asia countries [36]. Digitalization is reducing the cost of engaging in international trade, connecting businesses and consumers globally, helping to diffuse ideas and technologies and facilitating the coordination of global value chains. Choudhury (2020) assess the potential volume of digital trade in South Asia and estimated the possible loss of tax revenue incurred by this region during the last decade. For both South Asia and India, the results for actual import figure are found to be less than the estimated value [37].

The research on trade between China and BRI countries is a hot topic that gets a lot of attention. Based on previous studies, this paper makes further research on China-South Asia trade, including the trend and structure of China-South Asia trade, as well as its influencing factors. We investigate whether economic development, development gap, investment, distance, level of industrialization and accession to the BRI could affect the bilateral trade, including both economic and institutional factors.

### 2.5. Literature reviews

Above, the relationship between the Belt and Road Initiative, trade dependence and economic growth, as well as the influencing factors of trade and its role in specific commodity fields are summarized and analyzed in detail. Studies have shown that under the background of the Belt and Road Initiative, the trade between China and South Asia is affected by the economic growth of the two countries, which has opportunities and interdependence, and is also challenged by many aspects, such as politics. Based on the research on the influencing factors of international trade, we conclude that economic growth (GDP), geographical distance, market size, income and the degree of trade liberalization are the most important influencing factors of trade between China and countries along the Belt and Road Initiative.

Countries along the route have great differences in politics, economy, infrastructure and geographical location, which brings great risks and challenges to trade. Therefore, it is necessary to identify and analyze the trade influencing factors among countries along the "the belt and road initiative". At present, BRI is rarely used as a variable to introduce empirical models of the impact on South Asian trade. By establishing a trade gravity model, this paper analyzes the main influencing factors of trade between countries along the "the belt and road initiative", and estimates the trade potential of the two countries, thus providing certain theoretical support and reference for the trade quality.

## 3. Trade between China and South Asia

### 3.1. Summary of China-South Asia trade

The total value of imports and exports between China and South Asia has maintained rapid growth during 2004–2018. China’s imports to South Asia remained stable except that it fluctuated slightly under the influence of the global financial crisis from 2008 to 2010. Since 2013, China and most countries in South Asia have gradually reached a consensus on the "the belt and road initiative" issue. China and five South Asian countries-Sri Lanka (December 2014), Maldives (December 2014), Bangladesh (October 2016), Pakistan (May 2017) and Nepal (May 2017) signed the Memorandum of Understanding on "the belt and road initiative" cooperation. In July 2018, the Bhutanese government welcomed the positive measures of "the belt and road initiative". The Afghan government expressed the hope to participate more fully in the construction of "the belt and road initiative" in March 2022. Bilateral trade between China and eight South Asian countries has made great progress since 2013. Bilateral trade between China and eight South Asian countries totaled $96.252 billion in 2013, but by 2021, this figure has risen to $187.554 billion. The smooth implementation of the "the belt and road initiative" initiative in South Asia requires policy exchanges and strategic docking between China and countries in South Asia, especially in the core areas. For example, the "the belt and road initiative" strategy and Pakistan’s "Vision 2030" strategy should be organically combined to jointly promote the high-quality development of China-Pakistan Economic Corridor. To promote the convergence of the 21st Century Maritime Silk Road and Sri Lanka’s national development strategy, the two sides focused on three key areas: Colombo Port City Project, Hambantota Port Comprehensive Development and China-Sri Lanka Free Trade Area Negotiation. From the perspective of Balance of Trade (BOT), China enjoy a massive surplus in the trade with South Asia for a long time. Wang & Sun (2019) study on the trade between China and 8 South Asia countries. They also find the significant imbalances of trade between China and South Asia [38].

### 3.2. Trend of China-South Asia trade

**Fig 1:**
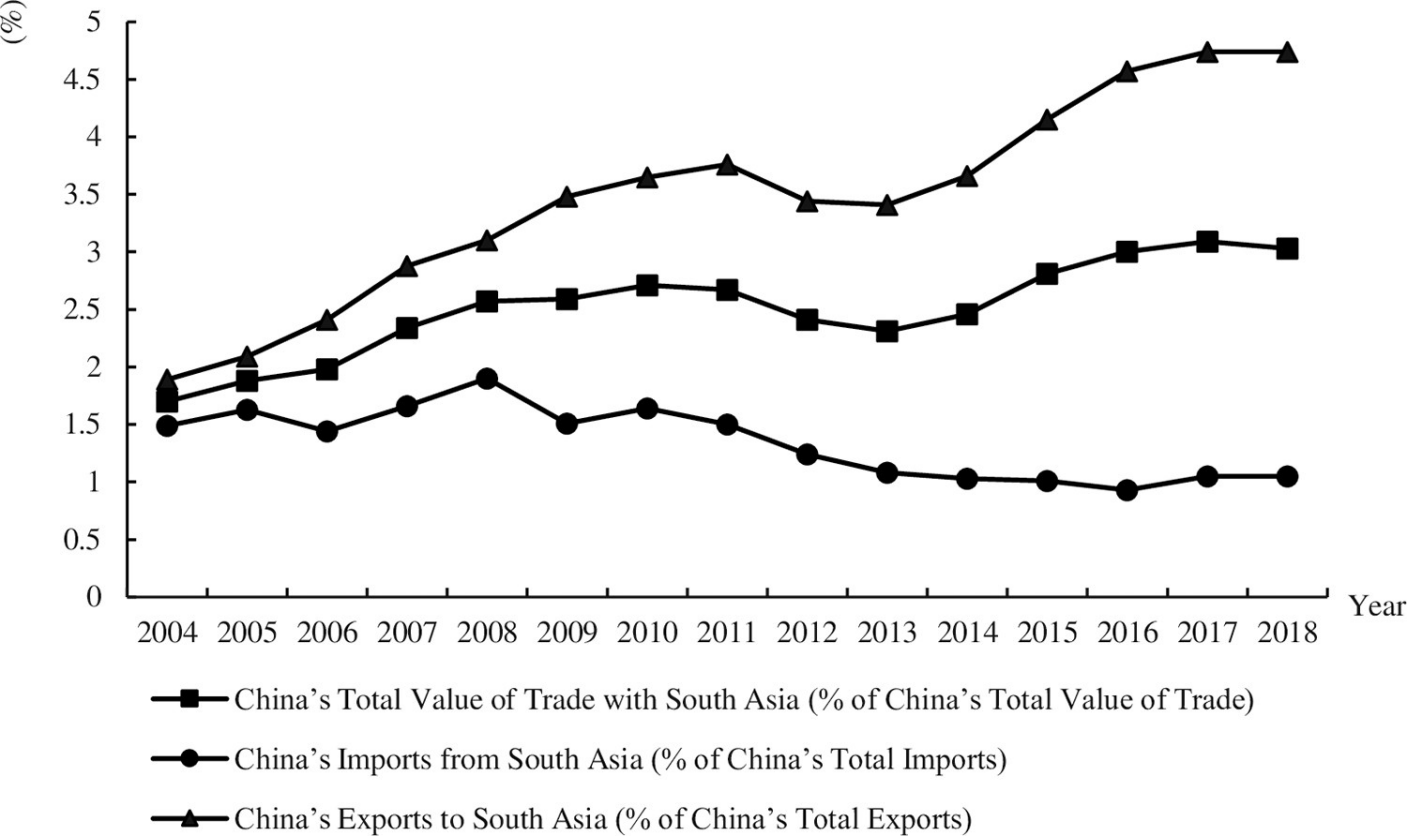
China’s trade with South Asia from 2004 to 2018.

Fig 1 shows China’s Trade with South Asia from 2004 to 2018.

The proportion of China’s total value of trade with South Asia to China’s total value of trade shows an increasing trend, raising from 1.7% in 2004 to 3.03% in 2018. There is a short term decreasing before 2013, but increasing significantly after 2013. Less than 2% of China’s total imports come from South Asia. And the proportion of China’s imports from South Asia to China’s total imports is decreasing. The proportion of China’s exports to South Asia increases from 1.89% in 2004 to 4.74% in 2018. This trend is particularly pronounced after 2013.

In 2021, ASEAN, the European Union, the United States, Japan and South Korea ranked in the top five. The growth rate of import and export trade of countries along the "the belt and road initiative" has obviously accelerated. According to the statistics of trading countries and regions, Asia is the largest trade zone in China, accounting for 51.59%. South Asia is the country with the highest population density in the world, but due to the limitation of economic development level, the eight South Asian countries are not China’s main trading partners. However, from the perspective of development, the economic and trade cooperation between South Asia and China is developing rapidly, and China’s trade status in South Asian countries has also been significantly improved.

**Fig 2:**
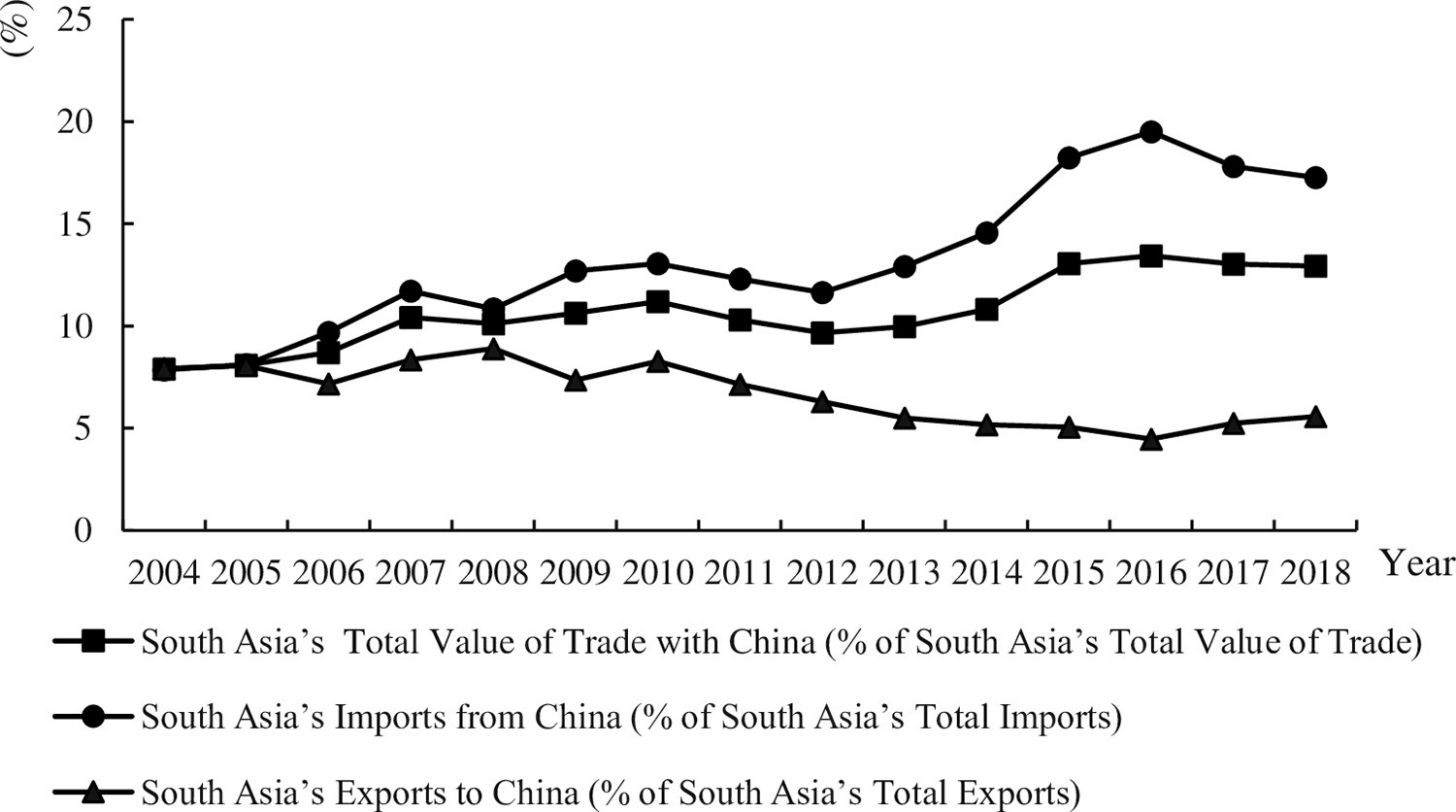
South Asia’s trade with China from 2004 to 2018.

Fig 2 shows South Asia’s Trade with China from 2004 to 2018. The proportion of South Asia’s total value of trade with China to South Asia’s total value of trade shows an increasing trend, raising from 7.89% in 2004 to 12.93% in 2018. The proportion of South Asia’s imports from China to South Asia’s total imports shows an increasing trend, especially in the three years after BRI was put forward. In 2016, approximately 20% of South Asia’s total imports come from China. The proportion of South Asia’s exports to China decreasing from 7.92% in 2004 to 5.56% in 2018. Therefore, China plays an important role in South Asia’s international trade. Compared to China, South Asia relies more on Chinese imports.

India is China’s largest trade partner among eight South Asia countries. The total value of trade between China and India has increased from 13,614 million US dollars in 2004 to 95,509 million US dollars in 2018, which has increased nearly 7 times in 15 years, China-India trade takes more than 60% of total value of trade between China and South Asia. The following is Pakistan, Bangladesh and Sri Lanka. The total value of trade between Pakistan and China is also increasing, takes 10% to 20% of the total value of trade between China and South Asia. The trade between China and Bangladesh expand rapidly, the value of trade raises significantly during 2004–2018. Both Nepal, Afghanistan, Maldives and Bhutan, accounting for less than 1% in China-South Asia trade.

### 3.3. Structure of China-South Asia trade

In this paper, we use Standard International Trade Classification (SITC) to classify the import and export commodities. According to Table 1, STIC-0 to STIC-4 are classified to primary commodity, STIC-5 to STIC-9 are classified to manufactured commodity.

**Table 1:** The standard international trade classification.

Classification	Category	SITC Class
Primary Commodity	Food & Live Animals	0
	Beverages & Tobacco	1
	Raw Materials	2
	Fuels & Lubricants	3
	Animal & Vegetable Oils	4
Manufactured Commodity	Chemicals	5
	Manufactured Goods	6
	Machinery & Transport Equipment	7
	Miscellaneous Manufactures	8
	Others	9

Data source: United Nations Statistics Division.

Table 2 shows that China’s exports to South Asia countries from 2004 to 2018. The commodities China exports to South Asia are mainly SITC-6, SITC-7 and SITC-8, which are manufactured commodities with relatively high value-added.

**Table 2:** The structure of China’s exports to South Asia countries.

Country	2004		2009		2014		2018	
	SITC Class	Proportion (%)	SITC Class	Proportion (%)	SITC Class	Proportion (%)	SITC Class	Proportion (%)
Afghanistan	7	59.82	7	59.19	7	44.48	7	35.98
	6	20.66	6	23.98	6	25.41	6	33.46
					8	22.90	8	22.02
Bengal	6	56.23	6	49.67	6	48.69	6	50.28
	7	27.67	7	25.03	7	25.65	7	25.09
Bhutan	7	83.70	6	57.69	8	34.71	8	51.56
			7	30.19	6	22.39	7	36.21
India	7	37.52	7	56.88	7	40.99	7	50.41
	6	24.16			5	22.15		
					6	21.42		
Maldives	8	30.18	8	34.83	7	43.26	7	36.16
	6	27.08	7	28.57	6	28.58	6	35.87
	7	25.74	6	28.35				
Nepal	8	32.49	8	44.77	8	77.63	7	36.61
	7	32.42	7	30.21			8	35.99
	6	21.81						
Pakistan	7	40.03	7	34.30	6	38.41	7	38.90
	6	21.38	6	34.17	7	32.65	6	32.25
Sri Lanka	6	57.46	6	41.67	6	39.75	6	43.76
	7	20.60	7	36.87	7	27.50	7	27.68

Note: Commodities accounting for more than 20% are listed in this table.

Table 3 presents the change in the structure of China’s imports from South Asia, showing that the structure of China’s imports from South Asia countries has changed significantly compared with China’s exports from 2004 to 2018. For example, China’s imports from Afghanistan, India and Sri Lanka are concentrated in SITC-2 before 2014, accounting for more than 50%. Imports of SITC-2 from India and Sri Lanka decrease to 20% approximately in 2012. In 2018, the import commodities were mainly SITC-6 and SITC-8.

**Table 3:** The structure of China’s imports from South Asia countries.

Country	2004		2009		2014		2018	
	SITC Class	Proportion (%)	SITC Class	Proportion (%)	SITC Class	Proportion (%)	SITC Class	Proportion (%)
Afghanistan	2	94.11	2	71.85	2	88.82	0	62.36
							6	20.64
Bengal	6	47.77	2	39.73	8	49.87	8	66.05
	2	25.10	6	27.30	6	20.72		
Bhutan	2	99.60	7	100.00	7	87.23	8	43.04
							1	21.59
India	2	64.57	2	64.79	6	46.07	6	36.49
					2	26.41	5	24.34
Maldives	5	69.59	8	68.51	7	78.84	3	84.51
			7	30.44				
Nepal	6	58.08	6	57.85	6	54.17	6	56.97
	4	25.05			2	35.75	8	28.49
Pakistan	6	88.53	6	81.15	6	72.34	6	61.31
Sri Lanka	2	51.24	2	50.22	8	31.27	8	34.80
					2	23.19		

Note: Commodities accounting for more than 20% are listed in this table.

Therefore, the structure of China’s exports from South Asia does not have significant change, which mainly focuses on the manufactured commodities. While the commodities China imports from South Asia has changed from primary commodities to manufactured commodities gradually.

## 4. Theoretical framework and methodology

### 4.1. Theoretical framework

The idea of the Gravity Model of Trade comes from Newton’s Laws of Universal Gravitation, which states that every particle attracts every other particle in the universe with a force which is directly proportional to the product of their masses and inversely proportional to the square of the distance between their centers. Since 1950s, Laws of Universal Gravitation was gradually introduced into the research on international trade, The Gravity Model of Trade highlights that geographical distance and economic size are the two basic factors determining the bilateral trade flows between the nations. The basic hypothesis of the Gravity Theory is size of the nations and trade flows have positive relationship, distance between nations and trade flows have negative relationship. The economic growth of a country can affect its market size. The larger the market size is, the greater the import and export demand is. The distance between the two countries can influence transportation costs in international trade.

Based on the Gravity Model of Trade and previous studies on the influential factors of international trade, we investigate whether economic growth, distance, international investment, national savings rate, level of industrialization and the accession to the BRI have influence on China-South Asia trade.

Economic growth of China and South Asia. GDP often used as the proper measure of the country’s potential trade. The GDP of the exporter measures productive capacity, while GDP of the importer measures absorptive capacity. Therefore, GDP is expected to be positively related to bilateral trade.

Geographical distance. Academia generally believes that the distance between two countries determines the cost of cargo transportation. The longer the distance is, the higher the transportation cost is.

International investment. With the China’s Go-Out Strategy, FDI has promoted the production efficiency of host countries through technology transfer, technology diffusion and spillovers, which has further promoted the trade cooperation.

Savings rate. A nation with lower savings rate indicates that the citizens tend to consume, while a nation with higher savings rate means that the citizens tend to consume in the future. The savings rate affects production and investment to some extent. Savings increase the capital stock by domestic investment, promote the infrastructure construction and economic development.

Industrial structure. The structure of a nation’s commodity trade depends on its industrial structure. The economic development of South Asia countries is relatively low. As mentioned above, the commodities China imports from South Asia changed from primary commodities to manufactured commodities, which also shows that South Asia countries are gradually organizing the agrarian economy into one focused on the mass production of goods and services. We choose the industrial value added (% of GDP) to measure the level of industrialization.

The impact of BRI on trade is another important factor we are interested in. BRI is composed of “Five Links” policy coordination, infrastructure connection, trade facilitation, financial capital flows and people-to-people exchanges.

### 4.2. Methodology

The traditional Gravity Model of Trade includes the total economic volume of bilateral trade and the distance between two countries. However, as mentioned above, there are other factors may affect the trade between China and South Asia. Therefore, we add some control variables to the model. The estimating equation is following:

$$\begin{array}{l} lnTR_{ict}=\alpha_{0}+\alpha_{1}lnGDP_{it}+\alpha_{2}lnGDP_{ct}+\alpha_{3}lnDIS_{ic}+\alpha_{4}GDPP_{ict}\\ +\alpha_{5}lnFDI_{ict}+\alpha_{6}DSR_{it}+\alpha_{7}IND_{it}+\alpha_{8}BRI_{it}+\varepsilon_{ict} \end{array}$$ (1)

$$\begin{array}{l} lnEX_{ict}=\beta_{0}+\beta_{1}lnGDP_{it}+\beta_{2}lnGDP_{ct}+\beta_{3}lnDIS_{ic}+\beta_{4}GDPP_{ict}\\ +\beta_{5}lnFDI_{ict}+\beta_{6}DSR_{it}+\beta_{7}IND_{it}+\beta_{8}BRI_{it}+\varepsilon_{ict} \end{array}$$ (2)

$$\begin{array}{l} lnIM_{ict}=\gamma_{0}+\gamma_{1}lnGDP_{it}+\gamma_{2}lnGDP_{ct}+\gamma_{3}lnDIS_{ic}+\gamma_{4}GDPP_{ict}\\ +\gamma_{5}lnFDI_{ict}+\gamma_{6}DSR_{it}+\gamma_{7}IND_{it}+\gamma_{8}BRI_{it}+\varepsilon_{ict} \end{array}$$ (3)

Where, *TR*_*ict*_ represents the total value of trade between China and country *i* in year *t*, *EX*_*ict*_ represents China’s exports to country *i* in year *t*, *IM*_*ict*_ represents China’s imports from country *i* in year *t*. *GDP*_*it*_ represents the GDP of country *i* in year *t*. *GDP*_*ct*_ represents China’s GDP in year *t*. *GDPP*_*ict*_ represents the difference of GDP per capita between China and country *i* in year *t*. *DIS*_*ic*_ represents the distance between the capitals of China and the capital of country *i*. *FDI*_*ict*_ represents the China’s investment to country *i* in year *t*. *DSR*_*it*_ is the first-order difference of savings rate of country *i* in year *t*. *IND*_*it*_ represents the industrial added value (% of GDP) in South Asia country *i* in year *t*. *BRI*_*it*_ is a dummy variable. If country *i* join the BRI or establish a free trade zone, the value is 1. Otherwise is 0. *α*_*0*_, *β*_*0*_, *γ*_*0*_ represents the constant term, *ε*_*ict*_ represents the error term. In order to eliminate heteroscedasticity, this paper applied natural log transformation on the condition of keeping the cointegration relationship of the original data. The description of explanatory variables and expected signs are shown in the Table 4.

**Table 4:** The description of variables and expected sign.

Variables	Description	Expected Sign
*GDP*_*ct*_	China’s GDP in year *t*	+
*GDP*_*it*_	GDP of country *i* in year *t*	+
*GDPP*_*ict*_	The difference of GDP per capita between China and country *i* in year *t*	-
*DIS*_*ic*_	Distance between the capital of China and the capital of country *i*	-
*FDI*_*ict*_	China’s investment to country *i*	+
*DSR*_*it*_	The first-order difference of Savings rate of country *i* in year *t*	+
*IND*_*it*_	The industrial added value (% of GDP) of country *i* in the year *t*	+
*BRI*_*it*_	Takes 1 if country *i* join the BRI or establish a free trade zone. Otherwise is 0.	+

### 4.3. Data

Due to some data of Bhutan is unavailable and the trade volume between the other seven countries and China accounts for more than 99% of the total value of China-South Asia trade, the data of the other seven countries are used. The data of trade volume between China and South Asia countries is from China’s National Bureau of Statistics. The data of GDP and the industrial added value (% of GDP) is from the World Bank Database. The data of saving rate is from International Monetary Fund Database. The distance between China and South Asia countries is from www.indo.com. Data on China’s direct investment in South Asia countries come from Statistical Bulletins of China’s Foreign Direct Investment. The data whether South Asia countries join the BRI or establish a free trade zone with China comes from the Belt and Road official website (www.yidaiyilu.gov.cn).

## 5. Results and discussion

### 5.1. Stationarity test

We test the stability of the data by using unit root test including common root-Levin, Lin & Chu, the individual root-ADF Fisher, the individual root-PP Fisher. All variables were integrated of order 1 at the 5% significance level, as shown in Table 5.

**Table 5:** Summary of unit root test in 1st difference.

Variable	Method		
	Levin, Lin & Chu t*	ADF—Fisher Chi-square	PP—Fisher Chi-square
*lnTR*	-5.9681 (0.0000)	54.0252 (0.0000)	58.6283 (0.0000)
*lnEX*	-6.55583(0.0000)	58.3042 (0.0000)	56.8630 (0.0000)
*lnIM*	-6.2229 (0.0000)	75.0010 (0.0000)	84.1833 (0.0000)
*lnGDP*_*it*_	-3.2313 (0.0006)	26.4870 (0.0224)	31.8317 (0.0042)
*lnGDP*_*ct*_	-8.8388 (0.0000)	50.2830 (0.0000)	45.0315 (0.0000)
*lnGDPP*_*ict*_	-6.7803 (0.0000)	44.9121 (0.0000)	44.5099 (0.0000)
*lnFDI*_*ict*_	-7.0894 (0.0000)	63.6529 (0.0000)	64.1329 (0.0000)
*SR*_*it*_	-10.749 (0.0000)	104.237 (0.0000)	114.326 (0.0000)
*IND*_*it*_	-6.8343 (0.0000)	67.9353 (0.0000)	76.2226 (0.0000)
*BRI*_*it*_	/	12.2087 (0.0022)	73.2521 (0.0000)

Notes: Prob.** in (). ** Probabilities for Fisher tests are computed using an asymptotic Chi-square distribution. All other tests assume asymptotic normality.

Kao Residual cointegration test could be performed to determine whether there was a long-term equilibrium relationship between variables. The results of the cointegration test are shown in Table 6.

**Table 6:** Results of Kao Residual cointegration test.

Series	t-Statistic	Prob.
*lnTR*, *lnGDP*_*it*_, *lnGDP*_*ct*_, *lnGDPP*_*ict*_, *lnFDI*_*ict*_, *SR*_*it*_, *IND*_*it*_, *BRI*_*it*_	-3.8773	0.0001
*lnEX*, *lnGDP*_*it*_, *lnGDP*_*ct*_, *lnGDPP*_*ict*_, *lnFDI*_*ict*_, *SR*_*it*_, *IND*_*it*_, *BRI*_*it*_	-4.2698	0.0000
*lnIM*, *lnGDP*_*it*_, *lnGDP*_*ct*_, *lnGDPP*_*ict*_, *lnFDI*_*ict*_, *SR*_*it*_, *IND*_*it*_, *BRI*_*it*_	-3.4760	0.0003

Notes: Null Hypothesis: No cointegration. Trend assumption: No deterministic trend.

### 5.2. Pool estimate

Fixed effect model is selected after we run the Hausmann test. Distance, which does not change with time, is eliminated in the regression process due to the limitation of panel data. The empirical results are shown in Table 7.

**Table 7:** Estimating results. Dependent variables: *lnTR*, *lnEX* and *lnIM*.

Explanatory variables	(1)	(2)	(3)
	*lnTR*	*lnEX*	*lnIM*
Constant	-19.7608***	-20.8344***	-50.0074***
	(6.7806)	(6.9105)	(11.6340)
*lnGDP*_*it*_	0.5983**	0.5920**	-0.8206*
	(0.2534)	(0.2475)	(0.4751)
*lnGDP*_*ct*_	0.5916	0.6325	2.9970***
	(0.4165)	(0.4263)	(0.6324)
*lnGDPP*_*ict*_	-0.1015	-0.1105	-1.2961***
	(0.2576)	(0.2651)	(0.3273)
*lnFDI*_*ict*_	0.0269	0.0294	0.1277***
	(0.0269)	(0.0273)	(0.0472)
*SR*_*it*_	0.0021	0.0018	0.0169**
	(0.0030)	(0.0031)	(0.0074)
*IND*_*it*_	0.0190*	0.0181	0.0016
	(0.0113)	(0.0115)	(0.0230)
*BRI*_*it*_	0.0140	-0.0101	0.1766*
	(0.0595)	(0.0593)	(0.1004)
AR(1)	0.5242***	0.5262***	0.7972***
	(0.1050)	(0.0885)	(0.0644)
Adjusted R-squared	0.9927	0.9934	0.9921
F-statistic	859.7754	940.6072	792.0930
Durbin-Watson stat	2.0414	2.0478	2.0420

Notes: Standard errors in (). The significance of the standard error

*** p <0.01

** p <0.05

* p <0.1.

The results show that influencing factors on the China-South Asia trade, China’s exports to South Asia and China’s imports from South Asia have significant differences.

On the China-South Asia trade as shown in column (1), the coefficients of *lnGDP*_*it*_ is positive and significant at 5% level, the coefficients of *IND*_*it*_ is positive and significant at 10% level, and the coefficients of AR(1) is positive and significant at 1% level, respectively, which indicates that economic growth in South Asia, the improvement of industrialization in South Asia countries, and the bilateral trade of last years can promote the bilateral trade. Therefore, we conclude that under the construction of the Belt and Road Initiative, the open economy of China-South Asia will be further deepened.

On China’s exports to South Asia as shown in column (2), the coefficients of *lnGDP*_*it*_ is positive and significant at 5% level, the coefficients of AR(1) is positive and significant at 1% level, respectively, which indicates that economic growth in South Asia, and China’s exports to South Asia of last years can promote China’s exports to South Asia. This was consistent with the findings of Zhao et al. (2019) and Chen & Xu (2018) [8, 9]. Thus economic growth in South Asia countries will expand demand, and based on the path-dependence such as import products from China as usual, this would increase China’s exports to South Asia further.

On China’s imports from South Asia as shown in column (3), most facts have significant effect and the same expected sign. The coefficient of *lnGDP*_*it*_ is negative and significant at 10% level, which is different from the expected sign. This is due to the restriction of economic development level, China mainly imports labor-intensive products from South Asian countries, because countries with lower economic development level have relatively few laborers, and the eight South Asian countries are not China’s main trading partners. The coefficients of *lnGDP*_*ct*_, *lnGDPP*_*ict*_, *lnFDI*_*ict*_ and AR(1) is significant at 1% level with the same expected sign. The economic growth in China, China’s invest, the common development of China-South Asia, the path-dependence such as import products from South Asia will encourage South Asia’s export to China. The coefficient of *SR*_*it*_ is positive and significant at 5%level. Therefore, the increase of savings rate of South Asia can expand domestic investment of South Asia countries and bring more opportunities to export goods for South Asia.

The coefficient *BRI*_*it*_ is positive and significant at 10% level in column (3), while not significant in column (1) and (2), This shows that the impact of the Belt and Road Initiative or establish a free trade zone at present is relied on China much more than on South Asia, which was consistent with the findings of Kong (2018) and Zhang (2019) [22, 26]. The BRI has many obstacles in South Asia, such as geopolitical competition in India, the terrorist activities in Afghanistan. Pakistan is trapped in the political turmoil with slow economic development.

To sum up, we concluded that, GDP, FDI and AR (1) have significant influence on China-South Asia trade, which means that economic growth, development gap, national savings rate and industrialization level are the main influencing factors of trade between China and South Asia. Among them, economic growth, savings rate and industrialization level of both sides have a significant positive impact, while the development gap between China and South Asia has a negative impact on trade between China and South Asia. The impact of BRI on China-South Asia trade is mainly reflected in China’s imports, which shows that the construction of the Belt and Road Initiative still needs to be deepened step by step.

## 6. Conclusions and policy implications

The trade volume between China and South Asia has been increasing gradually. But the trade imbalance is obvious—South Asia seriously depend on China’s imports. China mainly exports capital-intensive products to South Asia, while China imports labor-intensive products from South Asia. The economic growth, the development gap, national savings rate and the industrialization level have influence on the trade between China and South Asia. Among these influencing factors, economic growth of China and South Asia counties, savings rate and industrialization level of host countries have significant positive effect on the bilateral trade, while development gap has negative effect. However, the effect of BRI on China-South Asia trade is not significant. The following policy implications are proposed.

Promote China’s Going Out Strategy and encourage foreign direct investment. On the one hand, as China improve the value chain and increase the environmental awareness, many capital-intensive and labor-intensive industries, such as construction, manufacturing and energy, are shifting production abroad. Many Chinese multinationals bring advanced technology to invest in countries with rich resources and lower labor costs. On the other hand, expanding China’s investment in South Asia will facilitate technology transfer to domestic firms through spillover, which also promote domestic firms to make the strategic decision to adopt the advanced technology and stimulate innovation and creativity.

China and South Asia should deepen the cooperation of production. China has a relatively high level of industrialization. Deepening production capacity cooperation through building production bases and economic and trade cooperation zones can promote the industrialization of South Asia. The improvement of the industrialization expands the range of commodities of China-South Asia trade and optimize the trade structure.

Through combing the existing literature, although there are innovations, there are still limitations. For example, in terms of choosing influencing factors, although the research pursues the comprehensiveness of the theme, there may be other factors that have a certain impact on bilateral trade. In addition, the implementation of the Belt and Road Initiative policy is affected by many parties, and the implementation of specific projects and their impact on trade and economy need longer time and data to further prove and analyze. This study suggests that future research should continue to track and explore the trade influencing factors of South Asia and other countries and regions along the Belt and Road Initiative.

## Supporting information

S1 Data (XLSX)
